# Phleboviruses and the Type I Interferon Response

**DOI:** 10.3390/v8060174

**Published:** 2016-06-22

**Authors:** Jennifer Deborah Wuerth, Friedemann Weber

**Affiliations:** Institute for Virology, FB10-Veterinary Medicine, Justus-Liebig University, Giessen 35392, Germany; wuerthje@staff.uni-marburg.de

**Keywords:** phlebovirus, NSs protein, interferon, RIG-I, PKR

## Abstract

The genus *Phlebovirus* of the family *Bunyaviridae* contains a number of emerging virus species which pose a threat to both human and animal health. Most prominent members include Rift Valley fever virus (RVFV), sandfly fever Naples virus (SFNV), sandfly fever Sicilian virus (SFSV), Toscana virus (TOSV), Punta Toro virus (PTV), and the two new members severe fever with thrombocytopenia syndrome virus (SFTSV) and Heartland virus (HRTV). The nonstructural protein NSs is well established as the main phleboviral virulence factor in the mammalian host. NSs acts as antagonist of the antiviral type I interferon (IFN) system. Recent progress in the elucidation of the molecular functions of a growing list of NSs proteins highlights the astonishing variety of strategies employed by phleboviruses to evade the IFN system.

## 1. Introduction

The family *Bunyaviridae* contains five genera, among which the *Orthobunyavirus*, *Phlebovirus*, *Nairovirus*, and *Hantavirus* all contain species that are pathogenic to humans and animals, while the genus *Tospovirus* contains plant-infecting viruses [1]. According to the International Committee on Taxonomy of Viruses (ICTV), the genus *Phlebovirus* comprises more than 70 accepted members that are grouped into ten species complexes, namely Bujaru virus (BUJV), Candiru virus (CDUV), Chilibre virus (CHIV), Frijoles virus (FRIV), Punta Toro virus (PTV), Rift Valley fever virus (RVFV), Salehabad virus (SALV), sandfly fever Naples virus (SFNV), severe fever with thrombocytopenia syndrome virus (SFTSV), and Uukuniemi virus (UUKV), as well as unassigned viruses [2]. Phleboviruses were traditionally classified by serologic methods, but recently extensive efforts were undertaken to refine phlebovirus taxonomy by genome sequencing [3,4,5,6,7,8,9,10,11,12,13,14,15,16,17,18,19,20,21].

Phleboviruses can cause a wide spectrum of symptoms, ranging from mild febrile disease up to hemorrhagic fever and death [22,23]. RVFV, for example, causes disease in cattle, sheep, and other ruminants, with symptoms including hepatitis, hemorrhage, and abortion [24]. Humans exposed to RVFV can present febrile illness, but in 1% to 2% of the cases it can progress to retinitis with persisting visual impairment, meningoencephalitis or hemorrhagic fever, resulting in mortality of up to 20% in hospitalized patients [25]. Since its original description during an outbreak of RVFV in ruminants in Kenya in 1931 [26], periodic outbreaks have been observed throughout the African continent, accompanied by so-called ‘abortion storms’ in livestock populations and simultaneously occurring illness in humans. Notably, RVFV has spread to the Arabian Peninsula in 2000 [27].

Sandfly fever Sicilian virus (SFSV) and SFNV were isolated from foreign soldiers stationed in Italy during 1943 and 1944. In spite of a full recovery after the so-called ‘three-day’ or ‘Pappataci fever’, the febrile illness provoked by SFSV and SFNV can be incapacitating due to headaches, myalgia, and general malaise [22]. The strongly neurotropic Toscana virus (TOSV) was also isolated in Italy first. It is the predominant cause of meningitis or meningoencephalitis during the summer season in countries bordering the Mediterranean Sea [28]. Similarly to SFSV and SFNV, several phleboviruses in Central America have been isolated from febrile soldiers or patients, such as PTV, Chagres virus (CHGV), and Alenquer virus (ALEV) [17,29,30].

Despite their obvious capacity for causing human and veterinary disease, as well as potential associated economic losses, only few phleboviruses are adequately characterized in terms of their interaction with the mammalian host organism. In this review, we will attempt to provide an overview spanning both the current knowledge about the activation of the type I interferon (IFN) system by phleboviruses, as well as the broadening spectrum of their IFN-antagonistic strategies.

## 2. Phleboviruses—An Emerging Group of Arthropod-Transmitted Pathogens

Phleboviruses are arboviruses that are taxonomically divided into dipteran- and tick-borne viruses. Dipteran-borne phleboviruses are generally found in eponymous *Phlebotomus* sandflies [22,31], with RVFV representing an outlier that is associated with *Aedes* and *Culex* mosquitoes, and more promiscuous in its vector range. The specific vector species are thought to be predominantly responsible for the maintenance of the viruses by vertical (transovarial) transmission, the geographic distribution of the virus and the spatial and temporal occurrence of the specific disease. Given the increasing spread of competent vector species, concerns have been raised about the potential introduction of RVFV into new areas with both susceptible vectors and hosts, and potential consequences for the human population and massive economic loss caused among affected livestock [32,33].

The epidemiological potential of the phleboviruses has been underscored by the recent identification of two new members as the causative agents of severe human disease [23]. In rural regions of China, cumulative cases of a febrile illness accompanied by thrombocytopenia, leukocytopenia, multiple organ dysfunction, and a high case-fatality rate led to the discovery of a novel phlebovirus, SFTSV, transmitted by *Haemaphysalis longicornis* ticks [34,35,36,37,38]. Since its discovery, SFTSV and associated cases have also been reported from Japan and Korea [39,40,41]. In North America, nearly simultaneously-occurring cases of a similar set of symptoms were shown to be caused by a related, tick-transmitted phlebovirus termed Heartland virus (HRTV) [42,43]. Thus, while tick-borne phleboviruses were long thought to be negligible with respect to public health, the emergence of SFTSV and HRTV suggested that this perception needed reevaluation. As one result, the genome sequences of members of the Bhanja virus (BHAV) serogroup, which has been associated with febrile illness, were determined and re-classified into the tick-borne phlebovirus group [10,44].

Accumulating reports indicate novel associations of diseases with phleboviruses in the Mediterranean area, such as sandfly fever Turkey virus (SFTV) [45,46,47] and Adria virus (ADRV) [48], or describe still more novel phleboviruses, such as Granada virus (GRV) [49], Adana virus (ADAV) [3] and Medjerda Valley virus (MVV) [7], to name only a few examples.

## 3. Viral Replication in the Mammalian Host

**Figure 1:**
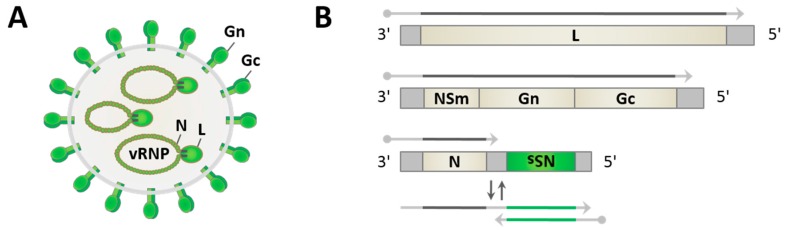
Prototypic phlebovirus virion and genome organization. (**A**) Virus particles contain the pseudocircularized tripartite single-stranded RNA genome, packaged into virus-sense RNPs (vRNPs) by nucleocapsid protein N and associated with the viral RNA-dependent RNA polymerase (RdRp) L, within a lipid envelope covered by heterodimers of glycoproteins Gn and Gc; and (**B**) the three viral genome segments large (L), medium (M) (both being purely negative-sense), and small (S) (ambisense) code for the structural proteins L, the Gn and Gc, and N, respectively. Viral mRNAs contain a 5′-cap (dot) and short heterogenous host-derived sequences. mRNAs transcribed from genomic RNAs are shown as grey arrows. The nonstructural protein NSs mRNA (green arrow) is synthesized from antigenomic RNA (two-colored arrow). Dipteran-borne phleboviruses also encode a nonstructural protein on the M segment (NSm).

Phleboviruses have spherical particles of approximately 100 nm diameter [23,50,51]. They are enveloped by a host-derived lipid membrane with the two viral glycoproteins Gn and Gc decorating the surface of the virus particle, and contain three distinct single-stranded RNA genome segments which are packaged into ribonucleoprotein particles (RNPs) by the nucleocapsid protein N and associated with the RNA-dependent RNA polymerase (RdRp) L (Figure 1A). The tripartite genome consists of the large (L), medium (M), and small (S) segments. The L and M segments are of negative polarity and code for the polymerase L and a polyprotein precursor spanning the two glycoproteins and the nonstructural protein NSm, respectively (Figure 1B). The S segment uses an ambisense coding strategy, i.e. it contains two genes with opposite polarities. The nucleocapsid protein N is thereby translated from a mRNA that is directly transcribed from the genomic S segment, whereas the nonstructural protein NSs mRNA is transcribed from the antigenomic S segment. Gene expression from the ambisense segments is regulated by an intergenic region (IGR), a sequence stretch that is proposed to form an irregular double-stranded RNA (dsRNA) structure [24], and by pentanucleotide transcription termination motifs [52,53,54]. The genome segments further contain conserved complementary oligonucleotide sequences at their 5′- and 3′-ends, allowing the formation of “panhandle” structures and the pseudocircularization of the RNPs [55].

Viral replication occurs entirely in the cytoplasm of infected mammalian host cells. Central features of the transmission from vector to host and the entry of phlebo- and other bunyaviruses has recently been reviewed elsewhere [56]. In short, after attachment of virus particles, uncoating is mediated by the fusion of the viral envelope with host membranes in the acidified compartments of the endocytic system [57,58]. Incoming RNPs then first serve as templates for primary transcription. To this end, the endonuclease domain [59,60] within the L protein cleaves host mRNAs 10–20 nucleotides downstream of the 5′-cap to use the resulting short fragments as primers for the synthesis of viral transcripts (cap snatching). Primary transcription is terminated prior to the segment termini via a specific sequence motif [52,53,54]. Phleboviral transcripts thus contain a 5′-cap and a short stretch of a heterogenous, host-derived sequence, but no poly(A) tail. Translation of viral proteins in the cytoplasm and at the endoplasmic reticulum (ER) is accompanied by cleavage of the polyprotein encoded by the M segment into Gn and Gc (and depending on the virus species, some other proteins e.g., NSm), heterodimerization of Gn and Gc, and their transport to the Golgi apparatus. For replication of the viral genome, the viral polymerase switches to primer-independent synthesis of full-length antigenomic RNA, which then, in turn, serves as a template for the synthesis of progeny genomic RNA. In a process called secondary transcription, these newly-generated genomes then produce even more viral mRNAs. Both the genomic and antigenomic RNA segments carry a 5′-triphosphate moiety and are packaged into RNPs. Assembly and budding finally take places at membranes of the Golgi apparatus, followed by release of virions via the secretory pathway.

The nonstructural proteins NSm and NSs are dispensable for viral replication [61,62,63,64]. sandfly-borne phleboviruses encode an NSm protein which may have a role in the regulation of apoptosis (as shown for RVFV [65]). The NSs protein is remarkable in its low conservation across the *Phlebovirus* genus compared to other viral proteins, with sequence similarities ranging only from approximately 10% to 30% [7,37]. As will be outlined below, the NSs protein is an important virulence determinant, acting as an inhibitor of the antiviral type I IFN system of the mammalian host [24,66,67].

## 4. The Type I Interferon System in RNA Virus Infection

Type I IFNs are cytokines that are produced by virus-infected cells [68]. In humans, there are thirteen IFN-α subtypes, a single IFN-β, and the less well-characterized IFN-ε, -τ, -κ, -ω, -δ, which activate the transcription of hundreds of IFN-stimulated genes (ISGs) [68,69]. Characterization of an ever-increasing number of ISGs shows that many of their products not only exert antiviral activity at every step of the viral replication cycle, but also possess antiproliferative and immunomodulatory functions.

The production of type I IFN is induced in response to conserved pathogen-associated molecular patterns (PAMPs), which are sensed by germline-encoded, so-called pattern-recognition receptors (PRRs). As PRRs of the cytoplasm, the RNA helicases retinoic acid-inducible gene 1 (RIG-I) and melanoma differentiation-associated protein 5 (MDA5) react to infection by distinct sets of RNA viruses [70]. RIG-I and MDA5 primarily recognize short 5′-triphosphate dsRNA, or long (preferentially of higher-order structure) dsRNA and its analogue polyinosinic:polycytidylic acid (poly(I:C)), respectively [71,72,73]. The prototypical RIG-I possesses two N-terminal caspase recruitment domains (CARDs), a central helicase domain and a C-terminal domain, and is kept in an auto-inhibited conformation by intramolecular interactions involving the CARDs and the helicase domains. Ligand binding by the helicase and C-terminal domains induces both ATP-dependent RIG-I oligomerization and a conformational switch, resulting in the exposure of the CARDs [73,74]. The latter then engage in K63-polyubiquitin-mediated homotypic CARD-CARD interaction with the adaptor mitochondrial antiviral signaling (MAVS) which in turn assembles prion-like fibrillary aggregates that are sufficient and necessary for the recruitment of tumor necrosis factor (TNF) receptor associated factor (TRAF) 2, 5, and 6 for downstream signaling [75,76]. The kinases TRAF family member-associated nuclear factor kappa-light-chain-enhancer of activated B cells (NF-κB) activator (TANK)-binding kinase 1 (TBK1) and inhibitor of kappa B kinase epsilon (IKKε) subsequently activate the transcription factor IFN regulatory factor 3 (IRF3) by phosphorylation, followed by its dimerization and nuclear accumulation, where it activates the production of type I IFN expression together with the transcription factors nuclear factor kappa-light-chain-enhancer of activated B cells (NF-κB) and activator protein (AP-1) [73].

Within the endosomal compartments, Toll-like receptor 3 (TLR3) recognizes viral dsRNA and poly(I:C), and signals via the adaptor Toll-interleukin 1 receptor (TIR) domain-containing adapter-inducing IFN-β (TRIF) to activate IRF3, NF-κB, and AP-1, and consequently induce the production of type I IFNs as well as inflammatory cytokines [77]. Further, recognition of single-stranded RNA by TLR7/8 and subsequent signaling via the adaptor myeloid differentiation primary response gene 88 (MyD88) results in the secretion of IFN-α, particularly by specialized plasmacytoid dendritic cells [78].

IFN-α/β bind to a common heterodimeric receptor, consisting of the subunits interferon-α/β receptor IFNAR1 and IFNAR2, on both infected and uninfected bystander cells. Signaling via the receptor-associated tyrosine kinases Janus kinase 1 (JAK1) and tyrosine kinase 2 (TYK2) leads to phosphorylation of Signal Transducer and Activator of Transcription 1 (STAT1) and STAT2, which then undergo heterodimerization and translocation to the nucleus. There, in a complex with IRF9, they bind to IFN-stimulated response elements (ISRE) within ISG promoters, finally resulting in the transcription of ISGs [68,69].

As the functions of the well characterized ISGs have been reviewed extensively elsewhere [68,79], only a few examples of antiviral ISGs will be described here. IFN-inducible transmembrane (IFITM) proteins interfere with fusion of the viral envelope at the plasma membrane (IFITM1) or in the endosomal pathway (IFITM2, 3) and, thus, the release of viral RNPs into the cytoplasm of infected cells [80]. The family of dynamin-like Mx GTPases are capable of restricting a wide range of viruses, presumably via trapping and missorting of incoming viral RNPs [81]. In contrast to other ISGs, Mx proteins are not expressed at low constitutive levels or in response to virus infection, but depend entirely on IFN signaling, rendering the abrogation of IFN induction and signaling an effective means of evading Mx activity. Protein kinase R (PKR) is expressed at low levels in an inactive form [82]. Binding of dsRNA results in PKR activation, leading to phosphorylation of its target eukaryotic initiation factor 2α (eIF2α) and, in consequence, the inhibition of the translation of both viral and cellular mRNA. PKR has also been implicated in NF-κB activation and the induction of apoptosis [83]. Interferon-induced protein with tetratricopeptide repeats (IFIT) proteins IFIT1, 2 and 3 are involved in translation inhibition and innate recognition of RNAs that lack proper 2′-*O* methylation or contain a 5′ ppp end [80,84].

Expression of the transcription factor IRF7 is also enhanced by IFN signaling. While the aforementioned activation of IRF3 leads to an initial wave of type I IFN secretion, including IFN-β and (in mice) IFN-α4, enhanced IRF7 expression and activation generates a second wave of type I IFN production which involves additional IFN-α subtypes [85].

In addition to direct antiviral effects of ISGs and the positive feedback loop via IRF7, type I IFN signaling also induces the production of a range of cytokines and chemokines, pro- and antiapoptotic factors, and affects multiple other signaling pathways. Through modulation of the differentiation and function of dendritic cells, T cells, natural killer (NK) cells, and B cells, type I IFNs shape the antiviral immune response beyond the initial innate immune response [68,79,86].

## 5. Activation of the Interferon System by Phleboviruses

Like other negative-strand RNA viruses, phleboviruses do not produce substantial amounts of dsRNA during infection [87,88]. As shown for RVFV, their naked virion RNA is, nonetheless, a strong activator of RIG-I due to the presence of the 5′-triphosphorylated dsRNA panhandle formed by the genome ends [89]. Moreover, also when packaged into RNPs, the RNA of RVFV particles can activate the RIG-I signaling pathway [90]. In fact, incoming RNPs already trigger RIG-I conformational switching and oligomerization, as well as IRF3 activation. Additionally, in vivo, the cytoplasmic RNA helicase/MAVS axis was demonstrated to be the primary IFN induction pathway for RVFV [91]. The in vivo role of TLRs, by contrast, is less clear. While Ermler et al. found for RVFV that neither the TLR7/8-MyD88 nor the TLR3-TRIF pathway play a significant role in IFN induction [91], Gowen et al. showed for PTV that TLR3 was activated and contributed to increased liver damage and mortality [92]. It remains to future studies to reveal whether these discrepancies are due to different experimental conditions or a differential ability of distinct phleboviruses to activate or inhibit TLR3.

Studies in a range of animal models suggested a protective effect of type I IFN in phleboviral infection. Treatment with synthetic type I IFN inducers, such as poly(I:C) or polyinosinic-polycytidylic acid, poly-l-lysine and carboxymethylcellulose (poly(ICLC)) in a prophylactic or therapeutic regimen was reported to protect mice and hamsters from lethal RVFV infection [93,94,95]. Similarly, administration of poly(I:C), poly(ICLC), or of IFN itself protect against PTV-induced liver damage and mortality in a mouse model [96,97,98], whereas treatment with IFN-neutralizing antibodies rendered otherwise resistant mice susceptible to PTV-associated death [99]. Several in vivo studies correlated the onset of type I IFN synthesis with increased survival after lethal RVFV challenge [100,101]. Lastly, mice deficient in IFN signaling are more prone to infections with RVFV and PTV [102,103]. Thus, induction of sufficient amounts of type I IFNs at an early point during infection is crucial for protective effects.

It is known that different viruses are targeted by distinct sets of ISGs [104,105]. Additionally, for phleboviruses, a number of inhibitory ISG products have been described (Table 1). Mx proteins drastically inhibit the replication of several phleboviruses, including RVFV, TOSV, and SFSV [106,107]. For human MxA it was shown that it sequesters RVFV N into large perinuclear complexes, thereby inhibiting primary and secondary transcription [108,109]. Replication of RVFV is also affected by IFITM2 and IFITM3, but not IFITM1, in accordance with their localization in the endocytic pathway and at the plasma membrane, respectively [110]. PKR is activated during phleboviral infection and can act as potent restriction factor [93,111]. Therefore, it is not surprising that PKR is targeted by different phleboviruses, as discussed below. Furthermore, IFIT proteins (mostly IFIT1 and IFIT2), long isoform of poly(ADP-ribose) polymerase 12 (PARP12L), 2'-5' oligoadenylate synthetase-like 2 (OASL2), and ISG15 influence the replication of RVFV [100,112,113]. The latter two ISGs are not upregulated in embryonic fibroblasts derived from a mouse strain with increased susceptibility to RVFV (MBT/Pas) and a generally decreased and delayed ISG response, compared to BALB/cByJ, C57BL/6J and 129/Sv/Pas mice. Small interfering RNA (siRNA)-mediated reduction of Oasl2 and ISG15, however, resulted in only slightly increased titers of recombinant NSs-deficient RVFV [100].

## 6. Viral Countermeasures

**Figure 2:**
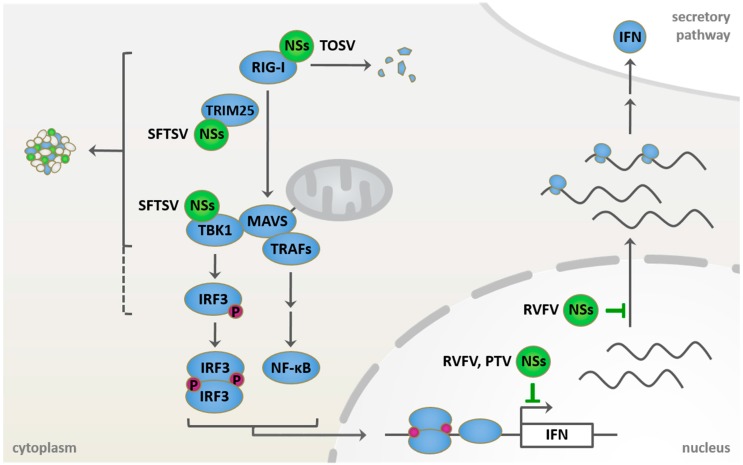
Known host targets of phleboviral NSs proteins in retinoic acid-inducible gene 1 (RIG-I) signaling and type I IFN induction. Incoming phleboviral RNPs are sensed by RIG-I, potentially leading to interferon induction via mitochondrial antiviral signaling (MAVS)-mediated activation of the transcription factors interferon regulatory factor 3 (IRF3) and nuclear factor kappa-light-chain-enhancer of activated B cells (NF-κB). NSs proteins, however, mediate the escape from the induction and the antiviral effects of the IFN system. Rift Valley fever virus (RVFV) NSs acts in the nucleus, where it blocks both the transcription and the export of host mRNAs. Toscana virus (TOSV) NSs localizes to the cytoplasm, where it interacts with and induces proteasomal degradation of RIG-I. Characteristic cytoplasmic structures are formed by severe fever with thrombocytopenia syndrome virus (SFTSV) NSs and serve as site of sequestration for several signaling factors of the RIG-I pathway. Punta Toro virus (PTV) NSs also inhibits host transcription.

As described above, phleboviruses are sensitive to IFN and an early induction of type I IFN seems to be a determinant of disease outcome in animal models. Furthermore, given the segmented nature of their genome, phleboviruses carry at least three RIG-I-activating moieties (5′ppp-dsRNA panhandle) per virus particle. Thus, in order to compensate for their stimulatory potential and to prevent or sufficiently delay a type I IFN response, they require highly efficient counterstrategies (Figure 2, Table 2 and Table 3).

The NSs protein of RVFV was the first to be identified as an IFN antagonist and still remains the most extensively studied phleboviral virulence factor. Comparative studies using the naturally-attenuated strain Clone 13 and the virulent RVFV isolate ZH548, as well as reassortants between these two viruses, showed that the S segment carries the determinant for attenuation and interference with IFN-α/β production in a murine model [102,114]. Since the S segment encodes the NSs and Clone 13 is a natural NSs deletion mutant, it was concluded that NSs confers an anti-IFN activity.

Although phleboviruses replicate exclusively in the cytoplasm, RVFV NSs is localized in the nucleus, forming characteristic filaments [115,116]. In contrast, Clone 13 contains a large in-frame deletion within the NSs open reading frame, resulting in a loss of 69% of the ORF [117]. Hence, Clone 13 NSs does not form nuclear filaments but instead is rapidly degraded [114]. The NSs of the RVFV wild-type strain ZH548 alone was then shown to almost completely block IFN-β promoter activation in response to poly(I:C), while Clone 13 NSs had no inhibitory effect [118]. Further, ZH548 did not affect IRF3 dimerization or nuclear accumulation, yet impaired IFN-β, NF-κB-driven, AP-1-driven, and even SV40 promoter activity, suggesting that RVFV NSs broadly inhibits both inducible and constitutive host cell transcription. Indeed, RVFV NSs targets the host mRNA synthesis machinery to induce a general cellular shutoff, including sequestration of general transcription factor II H (TFIIH) subunit p44 and, thus, prevention of TFIIH assembly [119]. In addition, NSs triggers the rapid proteasomal degradation of the TFIIH subunit p62 early in infection [120]. Proteomic analyses led to the identification of the F-box protein FBXO3 as host cell interactor of RVFV NSs [121]. F-box proteins are the substrate recognition component of modular E3 ubiquitin ligases of the Skp1, Cullin1, F-box (SCF) protein type [122], and FBXO3 was shown to be recruited by NSs to achieve TFIIH-p62 degradation [123]. The interaction with TFIIH-p62 thereby depends on a ΩXaV motif (where Ω: aromatic, X: any, a: acidic, V: valine) located in the C-terminal region of RVFV NSs [124]. Moreover, a nuclear mRNA export block was observed in RVFV NSs-expressing cells [125]. In contrast to these broadly-acting host cell shutoff mechanisms, RVFV NSs was also reported to specifically inhibit IFN induction by recruiting a transcriptional suppressor complex containing Sin3A associated protein 30 (SAP30) to the IFN-β promoter [126].

If not counteracted by viral measures, PKR has a strong restrictive effect on the replication of phleboviruses [93,111]. RVFV solves this problem by proteasomal degradation of PKR, thereby avoiding eIF2α phosphorylation and inhibition of translation [93,111]. Recent studies revealed that RVFV NSs recruits the F-box proteins FBXW11 and FBXW1 (also called beta-transducin repeat containing protein 1 (β-TRCP1)) as specific adaptors to mediate PKR degradation [127,128]. NSs thereby directly interacts with FBXW11/β-TRCP1 through a “degron” sequence [128]. Remarkably, this degron motif (DDGFVE) overlaps with the aforementioned ΩXaV motif (FVEV) necessary for TFIIH-p62 degradation, suggesting that RVFV NSs utilizes the very C-terminal part of the protein for the degradation of multiple host target factors, each time recruiting specific F-box proteins.

Infection of hamsters and mice with the PTV strain Adames is lethal, whereas PTV strain Balliet produces beneficial outcomes [103,129]. Reassortants between these two strains again identified the S segment genotype and NSs expression as correlates for lethality and suppression of type I IFN production [130]. Similar to RVFV, the NSs of PTV Adames has also been found to inhibit host transcription [131]. In contrast to RVFV NSs, however, PTV NSs does not form nuclear filaments or share the C-terminal ΩXaV motif of RVFV NSs [124]. A further difference between RVFV and PTV NSs is that the latter does not affect the levels of PKR [131,132].

In contrast to RVFV NSs, TOSV NSs localizes exclusively to the cytoplasm and does not affect cellular transcription [132,133], but inhibits IFN induction [134]. Instead, it has been shown to interact with RIG-I and trigger its proteasomal degradation [135]. Interestingly, binding of RIG-I and proteasomal degradation appear to be mediated by different regions of the NSs protein [136]. Also contrary to RVFV NSs, levels of TOSV NSs were found to be increased under MG132 treatment [132,135]. In line with this, C-terminally-truncated TOSV NSs mutants that were incapable of degrading RIG-I, but still able to bind RIG-I, were also detected at higher levels than the full-length protein [136], allowing speculations that TOSV NSs might be degraded along with its host target.

Peculiarly, while TOSV NSs efficiently inhibited IRF3 activation and IFN induction when expressed via transfection or from a recombinant RVFV, infection with the parental Italian TOSV isolate resulted in IRF3 activation, IFN-β induction and Mx expression [134]. A Spanish isolate, by contrast, was a potent IFN suppressor as expected from NSs action [137]. This discrepancy might be attributable to strain-specific differences in the kinetics of NSs accumulation.

Like RVFV NSs, TOSV NSs has also been observed to induce degradation of PKR in a proteasome-dependent manner [131,132].

Additionally, the NSs protein of the intermediately-pathogenic sandfly-borne SFSV possesses the capacity for inhibiting type I IFN induction [93,131]. The levels of PKR, however, are not affected [93,131,132].

The recent identification of tick-borne SFTSV as human pathogenic phlebovirus was quickly followed by a number of reports concerning the anti-IFN mechanism employed by its NSs protein. Type I IFN and ISGs were only moderately induced in SFTSV-infected cells, as observed by microarray analysis [138]. Indeed, SFTSV NSs was identified by several groups as inhibitor of IFN-β promoter activity, presumably acting at the level of TBK1 and IKKε [138,139,140,141].

SFTSV NSs neither forms nuclear filaments, nor is it diffusely distributed in the cytoplasm as described for the NSs proteins of sandfly-borne phleboviruses. Instead, it is concentrated in unprecedented cytoplasmic structures of granular appearance after both infection and transfection of a wide range of cell lines. Although these ‘viral inclusion bodies’ or ‘viroplasm-like structures’ seem to be subject to dynamic fission and fusion [139] and were found to be positive for the autophagosome marker microtubule-associated protein 1A/1B-light chain 3 (LC3), their formation was independent of autophagy-related protein 7 (Atg7), suggesting that they are not classical autophagosomes [140]. The early endosome RAS-associated protein Rab5 showed co-localization, but neither its presence nor canonical function were required for the formation of NSs inclusion bodies. Further analysis ruled out an association with ER, Golgi, mitochondria, peroxisomes, EDEMosomes, lysosomes and late endosomes, as well as aggresomes [139,140]. Furthermore, the inclusion bodies appeared to co-localize with lipid droplets and their formation associated with fatty acid synthesis [142].

Despite the open questions concerning biochemical composition and compartmental identity of the SFTSV NSs inclusions, it has become clear that they represent a site of sequestration and spatial isolation of multiple components of the RIG-I signaling pathway [139,140,141]. While all studies agree on TBK1 (and IKKε, where tested) as host interactors of SFTSV NSs, individual studies reported additional interactions with tripartite motif-containing protein 25 (TRIM25) (an E3 ubiquitin ligase involved in RIG-I signaling [143]), RIG-I [140], and IRF3 [139,141]. Remarkably, SFTSV NSs also sequesters transcription factors STAT1 and STAT2 into the inclusion bodies and inhibits STAT2 phosphorylation, thus interfering with their nuclear translocation, the stimulation of the interferon-stimulated response element (ISRE) promoter and, consequently, the induction of ISGs [144].

The non-pathogenic UUKV is the prototype of tick-borne phleboviruses. Its NSs is distributed throughout the cytoplasm [145] and has only a weak IFN-antagonistic effect [146]. Currently, there are no reports concerning the IFN-inhibitory capacity or action of the NSs proteins of HRTV and BHAV, despite their association with human illness.

Given the ambisense coding strategy of the S segment, the NSs would be expected to be expressed only late, after production of viral antigenomic RNA. This would represent a considerable disadvantage for the virus and is contradictory to the NSs-mediated effects that occur early after infection. This contradiction is resolved by the observation that antigenomic RNA segments are packaged into virions in both dipteran-borne RVFV and tick-borne UUKV [145,147,148]. Thus, the respective NSs proteins are directly produced during primary transcription, despite being encoded on the antigenomic RNA.

## 7. Conclusions and Future Directions

Phleboviruses are emerging arboviruses, causing human diseases ranging from mild febrile illness to severe cases of hemorrhagic fever or multiple organ dysfunction and death. Further, RVFV is associated with livestock epidemics and substantial economic losses. Within the genus *Phlebovirus*, the NSs protein is only weakly conserved in terms of its amino acid sequences or subcellular localization. Nevertheless, NSs proteins are highly conserved in their function as IFN antagonist, with their variety in sequence and localization being mirrored by a plethora of different molecular strategies. The diversity of IFN-antagonistic mechanisms of distinct phleboviruses tempts one to speculate whether a correlation between the NSs action and the degree of virulence exists.

Among sandfly-borne viruses, a common strategy of the more pathogenic members, such as RVFV and TOSV, seems the proteasomal degradation of host target factors that are involved in IFN induction or antiviral effector functions. Interestingly, while the NSs of the highly-virulent RVFV is not negatively affected, the NSs of intermediately pathogenic TOSV NSs seems to be susceptible to the proteasomal degradation machinery as well. For the highly pathogenic tick-borne virus SFTSV, the NSs protein sequesters a major fraction of the host factors involved in the RIG-I signaling pathway, as well as IFN signaling factors, into characteristic granular structures in the cytoplasm. This might suggest that broader action on multiple host cell functions, such as the general transcription block caused by RVFV or the deactivation of entire signaling chains, as seen for SFTSV, might be a correlate of increased phleboviral virulence.

Much of our current understanding of the functioning of the NSs protein has been achieved employing reverse genetics, such as the rMP12 and rZH548 rescue systems for the dipteran-borne RVFV [63,149,150,151], allowing the study of NSs-deficient mutants or chimeric viruses. Recently, reverse genetic systems were also established for the tick-borne phleboviruses UUKV and SFTSV [146,147]. It remains to future studies to further expand and specify the molecular characterization of the NSs proteins of both familiar and newly-emerging phleboviruses.

## Figures and Tables

**Table 1:** Interferon (IFN)-induced proteins acting as restriction factors for phleboviruses.

ISG	Affected Step in Replication	Affected Phleboviruses (Strains)	References
IFITM2, 3	uncoating	RVFV (ZH501, MP12)	[110]
Mx	primary and secondary transcription	RVFV (MP12, Clone 13), TOSV, SFSV	[106,107,108]
OASL2	?	RVFV (rZH548ΔNSs)	[100]
PKR	viral protein translation	NSs-deficient RVFV mutants (e.g., Clone 13)	[93,111]
IFIT1-3	viral protein translation	RVFV (Clone 13)	[113]
mPARP12	?	RVFV (MP12)	[112]
ISG15	?	RVFV (rZH548ΔNSs)	[100]

ISG: IFN-stimulated genes; IFITM: IFN-inducible transmembrane; OASL2: 2'-5' oligoadenylate synthetase-like 2; PKR: protein kinase R; IFIT: interferon-induced protein with tetratricopeptide repeats; mPARP12: murine poly(ADP-ribose) polymerase 12; RVFV: Rift Valley fever virus; TOSV: Toscana virus; SFSV: sandfly fever Sicilian virus.

**Table 2:** IFN-related host pathways targeted by diptera-borne phleboviruses.

Phlebovirus	Host Target	Mechanism	References
RVFV	TFIIH p44, XPB	sequestration	[119]
	TFIIH p62	proteasomal degradation by recruitment of a SKP1-FBXO3 E3-ubiquitin ligase complex	[120,123,124]
	SAP30	recruitment of suppressors to the IFN promoter	[126]
	mRNA export	unknown	[125]
	PKR	proteasomal degradation by recruitment of a SKP1-CUL1-FBXW11 E3 ligase complex	[93,111,127,128]
TOSV	RIG-I	proteasomal degradation	[134,135,136]
	PKR	proteasomal degradation	[132]
PTV	IFN induction	unknown	[131]
SFSV	IFN induction	unknown	[93,131]

PTV: Punta Toro virus; TFIIH: transcription factor II H; XPB: *xeroderma pigmentosum* type B; SAP30: Sin3A associated protein 30; RIG-I: retinoic acid-inducible gene 1.

**Table 3:** IFN-related host pathways targeted by tick-borne phleboviruses.

Phlebovirus	Host Target	Mechanism	References
SFTSV	RIG-I, TRIM25, TBK1/IKKε, IRF3	sequestration into cytoplasmic inclusion bodies	[138,139,140,141]
	STAT1, STAT2	sequestration into cytoplasmic inclusion bodies	[144]
UUKV	unknown	unknown	[146]

SFTSV: severe fever with thrombocytopenia syndrome virus; UUKV: Uukuniemi virus; TRIM: tripartite motif-containing protein; TBK1: (TANK)-binding kinase 1; IKKε: inhibitor of kappa B kinase epsilon; STAT: Signal Transducer and Activator of Transcription.
